# Depression and associated factors among older adults in Bahir Dar city administration, Northwest Ethiopia, 2020: Cross-sectional study

**DOI:** 10.1371/journal.pone.0273345

**Published:** 2022-08-23

**Authors:** Tamrat Anbesaw, Betelhem Fekadu

**Affiliations:** Department of Psychiatry, College of Medicine and Health Science, Wollo University, Dessie, Ethiopia; University of Huddersfield, UNITED KINGDOM

## Abstract

**Background:**

Depression is the most common psychiatric condition among older adults, and it goes unnoticed by individuals themselves and is under-diagnosed by clinicians due to the misconception that these are normal parts of aging. However, the problem is not properly addressed in Ethiopia. This study aimed to determine the prevalence and associated factors of depression among the older adults in Bahir Dar city.

**Methods:**

A community-based cross-sectional survey was conducted among 423 older adults in Bahir Dar city. A simple random sampling technique was used to select the study participants. Depression was assessed using a 15-item Geriatric Depression Scale (GDS). A multivariable logistic regression analysis was used to explore the potential determinants of depression among the participants.

**Results:**

The prevalence of depression among older adults was found to be 57.9% (95% CI: 53.2–62.6). This study showed that educational status with grades 5-8^th^ (AOR: 5.72, 95% CI: 2.87–11.34), and 9-12^th^ grade (AOR: 3.44, 95% CI: 1.59–7.41), income <2004 ETB (AOR = 1.89, 95% CI: 1.16–3.07), cognitive impairments (AOR: 3.54, 95% CI: 2.16–5.81), family history of mental illness (AOR:3.06, 95% CI: 1.03–9.04), and poor quality of life (AOR: 2.78, 95% CI: 1.74–4.46) were significantly associated with depression.

**Conclusion:**

The prevalence of depression among older adults was found to be huge. Having low educational status, low monthly income, cognitive impairments, family history of mental illness, and poor quality of life were associated with depression. Therefore, raising community awareness of mental health, increasing social participation, providing supportive counseling and routine screening of depressive symptoms are essential in combating depression among Bahir-Dar city older adults.

## Introduction

According to the Diagnostic and Statistical Manual of Mental Disorders (DSM-5), depression is a common psychiatric condition usually characterized by sadness, lack of interest, guilt or low self-esteem, disturbed sleep or food, exhaustion, and poor attention for at least two weeks [1]. Depression is the most frequent mental health disorder in the world, and it is a serious public health concern because it affects so many people including older adults [2, 3]. It has a prevalence rate of 10 to 55% [4].

Depression in older adults often goes untreated because people typically think that it is a normal component of the aging process and a natural reaction to chronic diseases, loss, and social conversion [2]. Depressive disorders afflict 10 to 20% of older individuals globally, affecting over 300 million people in 2015 as reported by WHO [5]. In addition to that, the aging population is on the rise in many countries of the world. By 2050, it is anticipated that 80% of the world older adults would live in low and middle-income nations, with the number of individuals aged 60 and above reaching 390 million [6]. When compared to their younger counterparts, older persons are more likely to face significant challenges in terms of financial loss, reliance on others, social deprivation, loss of self-worth, and functional limitations. They also have physical and mental health issues [7].

According to numerous studies showed, the prevalence of depression in older adults (aged 60 and above) in Chitradurga was 60% [8], while in Womberma District, Ethiopia, it was 45%. [9] South Africa 40% [10], North Indians 9.5% [11], Vietnam 6.9% [12], Egypt 44.4% [13], Malaysia systematic 16.5% [3], Singapore [14] 13.4%, Ethiopia (Harer) 28.5% [15], Ambo 41.8%, Chinese tertiary hospital 32.8% [16], Tanzania 21.2% [17], Greece 58.5% [18], systematic review conducted in Asian countries 38.6% [19] and Thailand 18.5% [20]. Suicide risk is higher among older adults when they are depressed. Suicide is the most common complication of depression, killing an estimated one million individuals each year [5]. The usage of health services by older adults increases as a result of depression, putting additional strain on the already overburdened healthcare system.

Genetic susceptibility, chronic disease and disability, pain, frustration with limitations in activities of daily living (ADL), personality traits (dependent, anxious, or avoidant), and adverse life events (separation, divorce, bereavement, poverty, social isolation) are all factors that increase depression risk in older adults, according to the WHO [7]. Also, many studies have shown a link between depression and various risk factors such as being a woman, living in a city, insomnia, older adults who are dependent on others, life stressors, lack of a spouse, lack of formal education, lower income, substance abuse, stressful life events, poor social support, more disability, lower life satisfaction, cognitive decline, employment status, and medical comorbidities [9–11, 13, 15, 16, 20–22].

Understanding the epidemiology of depression in older persons is crucial to lessen the harmful impact of depression on daily functioning and quality of life (QOL) [5]. Depression is largely ignored in healthcare strategy and planning in most underdeveloped nations, and mental health services receive only a small amount of funding [19]. In primary care settings, it is both underdiagnosed and undertreated. To the best of the authors’ knowledge, the burden of depression among the older adults in Ethiopia has not been adequately investigated, particularly among those living in Bahir Dar city. This gap may contribute to poor or inconsistent mental health care at the community level. As a result, this research was carried out to estimate the prevalence of depression in the older adults and to investigate the epidemiological factors that contribute to it.

## Method and materials

### Study design, study area and period

A community-based cross-sectional study was conducted in Bahir Dar city administration, which is located 565 km from Addis Ababa in North West of Ethiopia; the capital of Amhara regional state from June 1 to 30, 2020. According to the 2016–17 city administration report, the total population of Bahir Dar city administration is 266, 952; 124,396 males and 142,555 females. The city has nine sub-cities with 66,628 households. Among these, the age group of 60 years and above is estimated to be 11,034 (5003 male, and 6031 females). Those older adults are Shimbit (1670), Tana (1043), Fasilo (1200), Sefene selam (287), Gishabay (522), Shum ambo (417), Belay Zeleke (1591), and Ginbot-20 (4304). The health care service is provided by two specialized hospitals, one specialized and four primary private hospitals. There are also eleven health centers in Bahir Dar city administration. Information is taken from Bahir Dar city municipality.

#### Study population, inclusion, and exclusion criteria

All individuals older adults in the city aged 60 and above and residents of the city for at least six months were included, while older adults people who were severely ill, unable to communicate, and older adults with education below fifth grade were excluded from the study.

### Sample size determination and sampling technique

The sample size was calculated using the single population proportion formula with the assumption of a prevalence (P) of depression of 47.5% from a previous study [23] with a confidence limit of 5%. As a result, n = 384, with no requirement for a correction factor because the population size is more than ten thousand. The ultimate sample size was 423 after adding a 10% non-response rate. The Bahir Dar city administration urban division has nine sub-cities, one of which (Hidar 11) was excluded from the report due to insufficient data. Based on the population size, the final sample size was distributed proportionally to eight sub-cities. Participants included Shimbit (64), Tana (40), Fasilo (46), Sefene Selam (11), Gish Abay (20), Shum Abbo (16), Belay Zeleke (61), and Gimbot Haya (165). The sample frame (households with respective old ages) was obtained from health extension workers, and each household was then randomly selected using the lottery method. If more than one member fulfilled the criteria in one household one was selected using the lottery method. If no participants in the selected household fulfilled the criteria the next household was selected.

### Operational definition

#### Older adults

Are those aged is 60 and above [24].

#### Neurocognitive impairment

The MMSE score of ≤ 22 for those who attended less than eighth grade, ≤ 24 for those who attended grade nine to twelve, and ≤ 26 for those college/university graduates out of a total score of 30 [25].

#### Quality of life

Using WHOQOL-BREF 26-item index; a higher score denotes a higher quality of life [26].

#### The multidimensional scale of perceived social support scale

Any mean scale score ranging from 1 to 2.9 is considered as low, 3 to 5 moderate social support, and 5.1 to 7 as high social support [27].

#### Activities

Using Katz’s indicator of daily life independence, a total score of six shows independence, four suggests moderate independence, and two or less implies dependence [28].

#### Nutritional status

was assessed using the Mini Nutritional Assessment with the score ranged from 0–14 interpreted as (0–7) as malnourished, (8–11) at risk for malnutrition, and (12–14) as normal nutritional status [29].

#### Current substance use

Within the last three months, you have used at least one of a certain substance for non-medical purposes (alcohol, khat, tobacco, others) [30].

#### Ever use of a substance

Using at least one of any specific substances for a non-medical purpose at least once in a lifetime (alcohol, khat, tobacco, others [30].

#### Fast Alcohol Screening Test (FAST)

An overall total score of 3 indicates hazardous alcohol consumption [31].

#### Income

Using the World Bank poverty line cut point those who have an average monthly income of less than 2004 ETB (1.9 USD/day) taking 1$ = 35.16 ETB were taken as low income [32].

### Data collection tool

A structured interviewer-administered questionnaire was used to assess the sociodemographic factors, clinical related factors, behavioral and psychosocial factors. Geriatric Depression Scale (GDS-15) item was used to determine whether elderly people had depression or not. GDS-15 has undergone rigorous testing and validation in low- and middle-income countries including India and Nepal [33, 34].

The Royal College of Physicians, the British Geriatric Society, and the Royal College of General Practitioners all recommended this geriatric depression scale for screening depression in older adults [35]. A cutoff value of more than or equal to five was used to define depression [36]. The internal consistency (Cronbach alpha) of GDS-15 in this study was 0.86. Cognition status using standardized mini-mental state examination (MMSE) with a cut-off point as follows, no cognitive impairment (24–30), mild cognitive impairment (18–23), severe cognitive impairment (0–17) (39). It has been validated in Ethiopia for those with a formal education grade of fifth or higher, with different cut-off points depending on their level of education [25]. The specificity and sensitivity of MMSE were 77.8% and 78.7% respectively [37]. WHOQOL-BREF was used to assess the quality of life. It has four domains; physical, psychological, social, and environmental factors. It has internal consistency (Cronbach’s alpha > = 0.7) and has been translated into nine languages [26]. The six-question mini-nutritional assessment short form has been validated in Ethiopia. Cronbach’s alpha was 0.65, with 80.1 percent sensitivity and 72.5 percent specificity (54). Katz’s index of daily life independence consists of six questions, each worth one point. Cronbach’s alpha was found to be 0.83, with strong test-retest and inter-rater reliability [38]. History of mental illness presence of chronic medical illness, and substance-related factors were assessed with yes/no questions, but alcohol drinking was assessed by using FAST.

### Data collection procedure

Data was collected through face-to-face interviews by trained data collectors. The data were collected from study participants by face-to-face interviews from house to house. The questionnaire was prepared in English and then translated into the local language (which is Amharic) by a language translator and translated back to English to ensure its understandability and consistency before the actual data collection. The training was given for the supervisor and data collectors by the principal investigator for two days duration on the methods of data collection and the detail of the questionnaire. Data were collected by four psychiatric nurses who currently work in health centers and was supervised by Masters of Sciences degree holder in mental health. A pretest was conducted on 21(5%) to check the understandability of the questionnaires. The collected data were reviewed and checked for completeness before data entry.

### Data analysis

The completed questionnaire was manually checked for completeness. Data were coded and entered into Epi data version 4.6 and, then exported to SPSS- 26 version for analysis. Descriptive and summary statistics were used to explain the population concerning the relevant variables. The bivariate logistic analysis was done to determine the association between the outcome and explanatory variables. Variables with p less than 0.25 in the bivariate analysis were entered into multivariate analysis. Multivariable logistic regression analysis was employed to control for possible confounding effects and to determine the presence of a statistically significant association between independent variables and outcome variables. The model of fitness was checked by Hosmer and Lemeshow goodness and a p-value less than 0.05 was considered statistically significant and the strength of the association was presented by an odds ratio of 95% C.I.

### Ethical consideration

Ethical clearance was obtained from the Institutional Review Board of Bahir Dar University. Study participants were informed about the procedure, the significance of the study, risks, and benefits associated with the study. Written Informed consent was obtained from participants who participated in the study. Each respondent was informed about the objective of the study and all data obtained from them was kept confidential by using code instead of any personal identifier which was used only for the study. The information was not disseminated without the respondent’s permission. The information provided by the participants was exclusively utilized for the study. Those older adults who reported depression were immediately referred to mental health facilities for further evaluation and management.

## Results

### Socio-demographic characteristics of participants

A total of 423 older adult individuals participated in this study (100% of response rate). The mean age (SD) of the participants was 66.01(±5.88), 58.9% were males. The majority of the participants 52.2% had a spouse. Almost two-thirds of the participants 66.6% were Orthodox Christian followers and the majority 86.3% were Amhara in their ethnicity. Regarding educational level, 61.5% were from grade five to eight. Around one-third, 36.6% were housewives followed by retired 27.0%. From the participants, 62.9% participants reported that their average monthly income was ≥2004 ETB, and 63.6% were living with their family (Table 1).

**Table 1:** Socio-demographic characteristics of older adults in Bahir Dar city administration, northwest, Ethiopia, 2020 (n = 423).

Variables	Categories	Frequency(n = 423)	Percent (%)
Age in years	60–64	191	45.1
	65–69	127	30.0
	70–74	69	16.3
	75–79	18	4.3
	80 and above	18	4.3
Sex	Female	174	41.1
	Male	249	58.9
Marital status	Has spouse	221	52.2
	No spouse	202	47.8
Religion	Orthodox	282	66.6
	Muslim	117	27.7
	Protestant	16	3.80
	Catholic	8	1.90
Ethnicity	Amhara	365	86.3
	Oromo	14	3.3
	Tigre	27	6.4
	Gurage	17	4
Educational status	5-8^th^ grade	260	61.5
	9-12^th^ grade	99	23.4
	College and above	64	15.1
Occupational status	Governmental employee	17	4.0
	Merchant	105	24.8
	Housewife	155	36.6
	Retired	114	27.0
	Others*	32	7.6
Monthly income	<2004ETB	157	37.1
	≥2004 ETB	266	62.9
Current living condition	Alone	92	21.7
	Relative	62	14.7
	Family	269	63.6

Key: * Farmer, Jobless.

### Clinical and substance-related factors of the participants

According to this study finding, 42.1% of respondents had neurocognitive impairment. More than half, 51.1% of participants had a comorbid medical illness, such as hypertension 30.7%, HIV/AIDS 6.1%, cardiac 5.9%, diabetes 18%, and others 2.6%. Of the participants, 46.8% currently used medication and 8.0% had reported a family history of mental illness. Among the respondent, 11.8% of the respondents had a history of head trauma and 53.4% were normal nutritional status (Table 2).

**Table 2:** Clinical characteristics of older adults people in Bahir Dar city administration, northwest, Ethiopia, 2020 (n = 423).

Variables	Categories	Frequency(n = 423)	Percent (%)
Cognitive impairments	Yes	178	42.1
	No	245	57.9
History of chronic medical illness	Yes	216	51.1
	No	207	48.9
Hypertension	Yes	130	30.7
	No	87	20.6
HIV/AIDS	Yes	26	6.1
	No	192	45.4
Cardiac	Yes	25	5.9
	No	192	45.4
Diabetes	Yes	76	18.0
	No	141	33.3
Others*	Yes	11	2.6
	No	207	48.9
Medication currently in use	Yes	198	46.8
	No	225	53.2
Family history of mental illness	Yes	34	8.0
	No	389	92.0
History of head trauma	Yes	50	11.8
	No	373	88.2
Nutritional status	Malnourished	32	7.6
	Risk	165	39
	Normal	226	53.4

Others*: Epilepsy.

**Fig 1:**
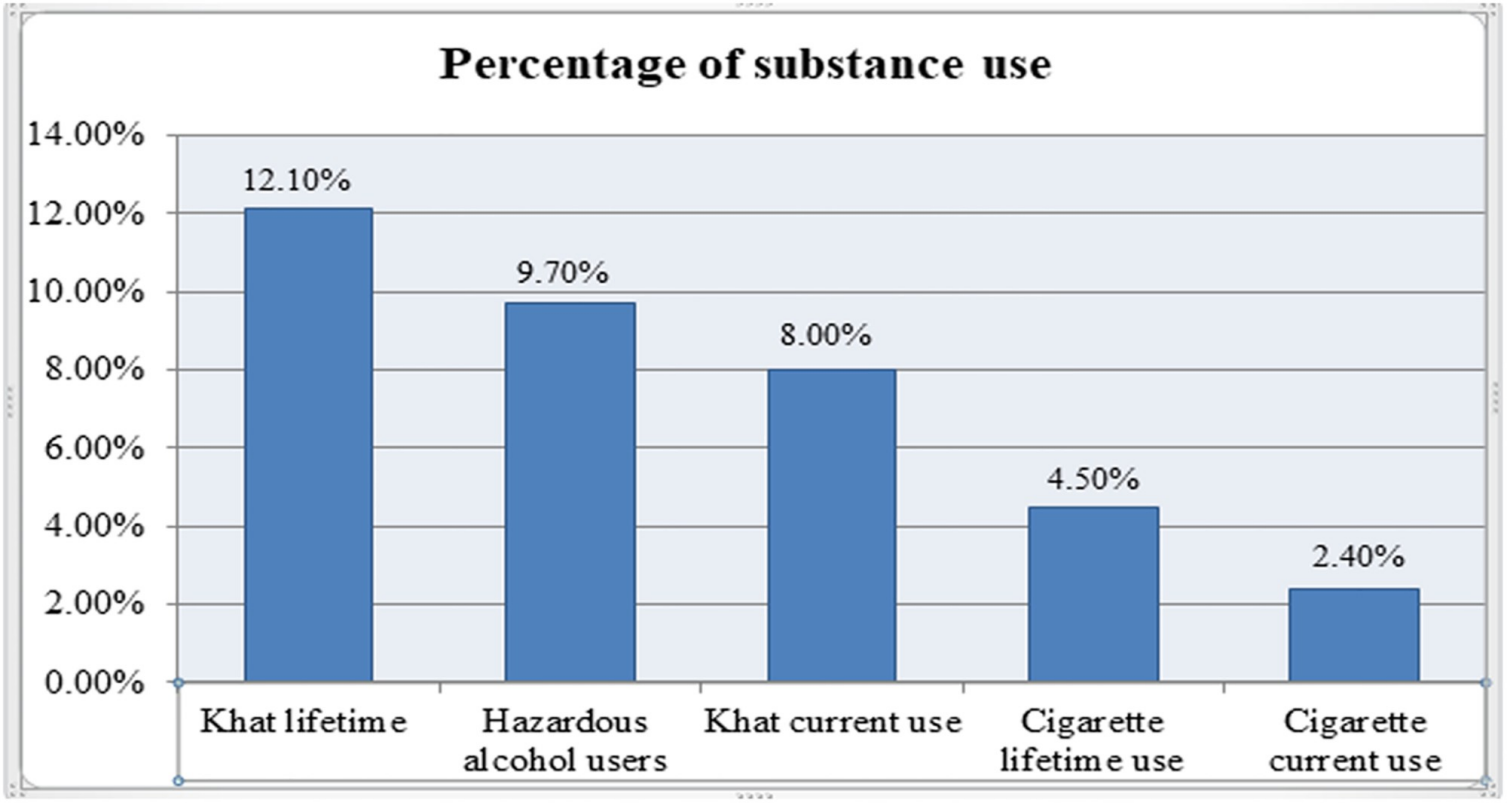
Ever and current substance use among older adults people in Bahir Dar city administration, northwest, Ethiopia, 2020 (n = 423).

Regarding the use of the substance, about 9.7% of them were hazardous alcohol users. Of the participants, 8% and 2.4% were using khat and cigarettes within the past three months respectively (Fig 1).

### Psychosocial characteristics of respondents

Regarding the psychosocial factors of respondents around 47.5% of them had poor quality of life. From the participant, 13.0%, 13.2%, and 13.5% had low family social support, low friend social support, and low other social support respectively (Table 3).

**Table 3:** Psychosocial characteristics of older adults people in Bahir Dar city administration, northwest, Ethiopia, 2020 (n = 423).

Variables	Categories	Frequency(n = 423)	Percent (%)
The activity of daily living	Dependent	9	2.1
	Moderate	14	3.3
	Independent	400	94.6
Quality of life	Poor quality	201	47.5
	Good quality	222	52.5
Family social support	Low support	55	13.0
	Moderate support	136	32.2
	High support	232	54.8
Friend social support	Low support	56	13.2
	Moderate support	213	50.4
	High support	154	36.4
Significant other social support	Low support	57	13.5
	Moderate support	150	35.5
	High support	216	51.1

### Prevalence of depression and associated factors among older adults people

**Fig 2:**
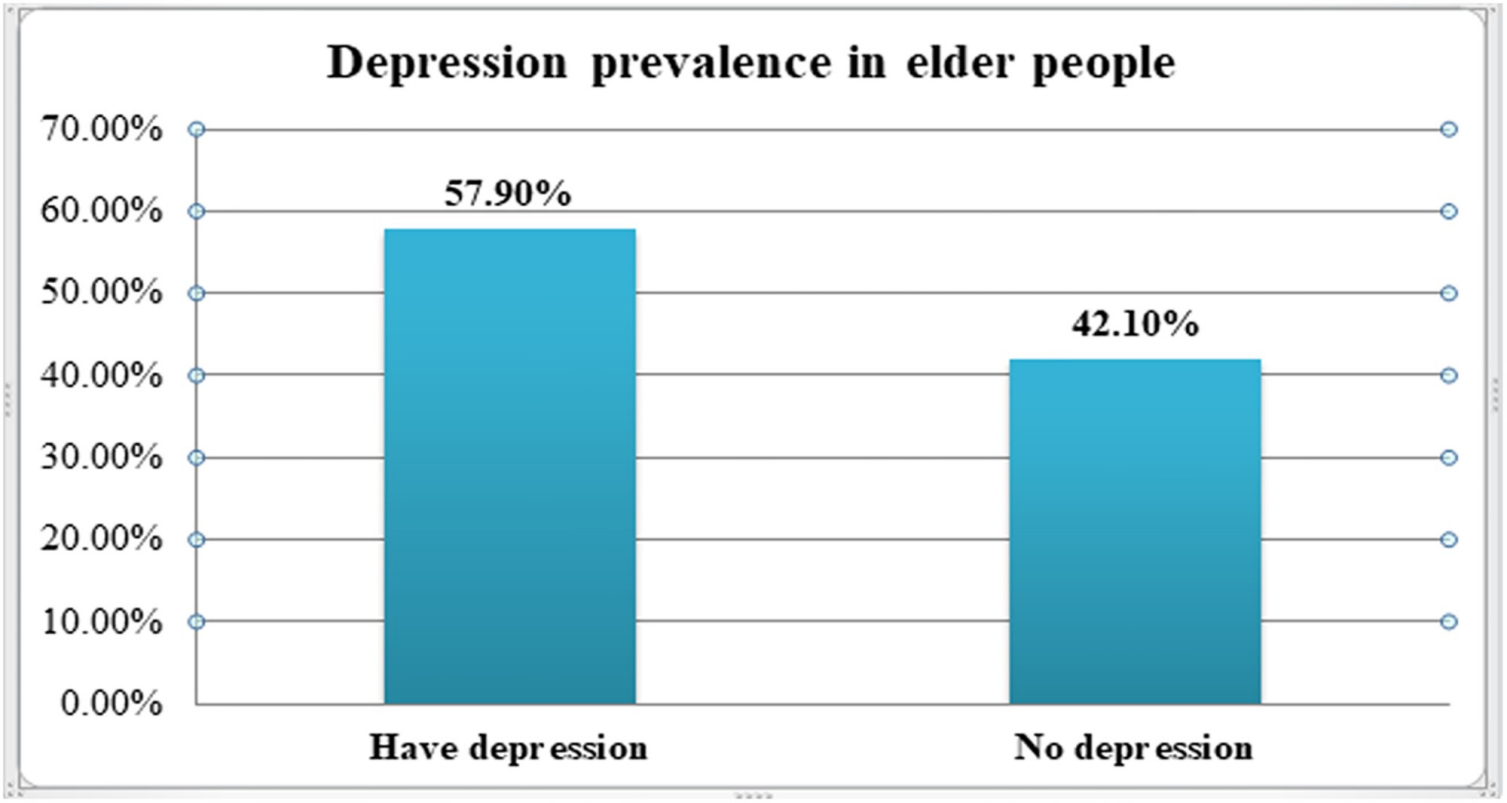
Prevalence of depression in older adults people in Bahir Dar city administration, northwest, Ethiopia, 2020 (n = 423).

In this study, the overall prevalence of depression among older adults people in Bahir Dar city was 57.9% (95% CI: 53.2,62.6) (Fig 2).

Factors such as marital status, educational status, occupational status, monthly income, current living condition, cognitive impairment, history of chronic medical illness, medication currently in use, family history of mental illness, and quality of life were significantly correlated (P < 0.25) in bi-variable analysis. Among these, variables such as educational status, income, cognitive impairment, family history of mental illness, and poor quality of life were significantly associated with depression in multivariable analysis.

Older adults whose educational status was grades 5-8^th^ were nearly six times (AOR: 5.72, 95% CI: 2.87–11.34), and 9-12th grade were 3.44 times (AOR: 3.44, 95% CI: 1.59–7.41) more likely to develop depression compared to college and above. Older adults with a monthly income of <2004 ETB were nearly 2 times more likely to have depression as compared to participants with an income of <2004 ETB (AOR = 1.89, 95% CI: 1.16–3.07). Older adults who had cognitive impairments were 3.54 times more likely to develop depression compared with their counterparts (AOR: 3.54, 95% CI: 2.16–5.81), and, who had a family history of mental illness were also three times more likely to have depression compared to those who had no family history of mental illness (AOR:3.06, 95% CI: 1.03–9.04). Finally, older adults with poor quality of life were 2.78 times more likely to develop depression compared to good quality of life (AOR: 2.78, 95% CI: 1.74–4.46) (Table 4).

**Table 4:** Bivariate and multivariable logistic regression analysis results of depression in Bahir Dar city administration, northwest, Ethiopia, 2020 (n = 423).

Variables	Category	Depression		COR(95%C.I)	AOR(95%C.I)	P-values
		Yes(n)	No(n)			
Marital status	No spouse	145(71.8%)	57(28.2%)	3.08(2.05,4.62)	1.49(0.92,2.44)	0.108
	Has spouse	100(45.2%)	121(54.8%)	1	1	
Educational status	5-8^th^ grade	176(67.7%)	84(32.3%)	5.79(3.14,10.69)	5.72(2.87,11.34)	**<0.001** *
	9-12th grade	52(52.5%)	47(47.5%)	3.06(1.55,6.04)	3.44(1.59,7.41)	**0.002** *
	College and above	17(26.6%)	47(73.4%)	1	1	
Occupational status	Government employee	6(35.3%)	11(64.7%)	1	1	
	Merchant	52(49.5%)	53(50.5%)	1.79 (0.62, 5.22)	0.96(0.25,3.66)	0.949
	Housewife	100(64.5%)	55(35.5%)	3.333(1.170,9.50)	1.33(0.35,5.04)	0.669
	Retired	61(53.5%)	53(46.5%)	2.11(0.73, 6.09)	1.41(0.37,5.28)	0.612
	Others**	26(81.3%)	6(18.8%)	7.94(2.09, 30.13)	1.71(0.34,8.63)	0.516
Monthly income	<2004ETB	112(71.3%)	45(28.7%)	2.49(1.63,3.79)	1.89(1.16,3.07)	**0.01** *
	≥2004 ETB	133(50.0%)	133(50.0%)	1	1	
Current living condition	Alone	65(70.7%)	27(29.3%)	2.22(1.33, 3.69)	0.83(0.39,1.75)	0.634
	Relative	40(64.5%)	22(35.5%)	1.67(0.94, 2.97)	0.46(0.21,1.03)	0.061
	Family	140(52.0%)	129(48.0%)	1	1	
Cognitive impairment	Yes	140(78.7%)	38(21.3%)	4.91(3.16,7.62)	3.54(2.16,5.81)	**<0.001** *
	No	105(42.9%)	140(57.1%)	1	1	
History of chronic medical illness	Yes	139(64.4%)	77(35.6%)	1.72(1.16,2.54)	1.17(0.74,1.86)	0.492
	No	106(51.2%)	101(48.8%)	1	1	
Medication currently in use	Yes	130(65.7%)	68(34.3%)	1.83(1.23,2.71)	1.04(0.36,3.02)	0.943
	No	115(51.1%)	115(51.1%)	1	1	
Family history of mental illness	Yes	29(85.3%)	5(14.7%)	4.64(1.76,12.25)	3.06(1.03,9.04)	**0.043** *
	No	216(55.5%)	173(44.5%)	1	1	
Quality of life	Poor quality	153(76.1%)	48(23.9%)	4.50(2.96,6.85)	2.78(1.74,4.46)	**<0.001** *
	Good quality	92(41.4%)	130(58.6%)	1	1	

*Statistically significant at P-value < 0.05, COR, Crude odds Ratio, AOR, Adjusted odds Ratio, 1 = reference category, Chi square = 8, Hosmer Lemeshow goodness-of-fit 0.42, degrees of freedom = 8 and,

** Farmer, Jobless.

## Discussion

The high prevalence of depression in this study 57.9% [95% CI: 53.2% − 62.6%], may be the indicative of a high burden due to depression among older adults in the community. The finding was congruent with that of a community-based cross-sectional study done in Chitradurga, India (60%) [8], Heraklion, Greece (58.5%) [18], Portugal (61.4%) [39], and India (53.75%) [40]. This result was lower than those found in studies in Greece (84.3%) [41], Vietnam (66.9%) [12], urban, India (75.5%) [42], and Beni Suef, Egypt (89.7%) [43]. This disparity in prevalence could be related to differences in the tools employed to measure depression. For instance, in urban India, the 30-item GDS is used to screen for depression, whereas in Vietnam, the Zung self-rating depression scale is used to screen for depression [12]. Furthermore, the heterogeneity in the prevalence of depression among older adults could be explained by differences in study design, sampling procedure, socioeconomic-demographic characteristics, geographical location, and cultural differences.

However, this study finding was higher than study done in North Indian (9.5%) [11], Malaysian (16.5%) [3], Tanzania 21.2% [17], a systematic review conducted in China (38.6%) [19], Thailand (18.5%) [20], Ambo, Ethiopia (41.8%) [21], Singapore 13.4% [14], China 32.8% [16], and Womberma district, Ethiopia (45%) [9]. This variation might be due to social-cultural, economic disparities, and the heterogeneity in the classification of depression, i.e., they utilized a GDS-15 score of 6 and above to define depression, which could lead to an underestimating of depression prevalence in Chinan [19]. Another probable reason is the difference in assessment technique; in Singapore, depression was assessed using the Geriatric Mental State (GMS) instrument [14]. Whereas, in our study, depression was assessed using the Geriatric Depression Scale item 15 (GDS-15) tool. Additionally, the disparity could be due to a difference in the study participants; in Ambo, the majority of the participants were males, which was found to be less likely to be depressed than females in the study [21]. Furthermore, according to some studies, people in developed countries have easier access to mental health care and support before they experience problems.

Regarding the associated factors, older adults whose educational status grades 5-8^th^ were nearly 6 times and 9-12th grades were 3.44 times more likely to develop depression compared to college and above. This finding was in agreement with different studies in Ethiopia (Harer) [15], Malaysia [3], India (Punjab) [22], Egypt [44], and Thailand [20]. Depressive symptoms are linked to educational attainment, and depression can be influenced by a variety of socioeconomic factors. In lower levels of educational achievement, there is no simple strategy to improve the health and economic success of a nation.

Older adults with a monthly income <2004 ETB were nearly 2 times more likely to have depression as compared to participants with an income of ≥2004 ETB. Similar to a finding of different studies reported in Asia (Myanma) [45], North Indians [11], and Portugal [46]. This is the finding that low-income people have more difficult getting healthy services and care, which has been associated with higher levels of depression. McCall and colleagues’ findings in the United States supported prior studies that connected low income to a higher prevalence of depression [47].

Older adults who had cognitive impairments were 3.54 times more likely to develop depression compared with their counterparts. This was supported by the study conducted in Ethiopia (Harer) [15] and Chinese tertiary hospitals [16]. According to Ismail’s meta-analysis, depression is common among people with mild cognitive impairment (MCI), with a pooled prevalence of 32% [48]. Depression can result from problems with attention and working memory, as well as changes in sleep patterns and social isolation due to cognitive impairment [49]. Furthermore, MCI shares some of the same characteristics as late-life depression in terms of brain structure changes [50].

A family history of mental illness was also a predictor of depression. When compared to respondents who did not have a family history of mental illness, those populations who had a family history of mental illness were three times more likely to be depressed. This could be explained by the fact that mental illness is inherited, that families are stigmatized, and that there are various types of burdens on family members in terms of financial expenses and providing care for the patient, as well as the offspring may be stressed and worried about their parent’s health condition, all of which could increase the risk of depression [51].

Finally, older adults with poor quality of life were 2.78 times more likely to develop depression compared to good quality of life. This matches research from North Indians [11], Chinese tertiary hospitals [16], and Portugal [46]. Our findings show that older people with depression are more likely to report poor quality of life. In a review article comprising 74 studies, Sivertsen and colleagues came to the same conclusion, finding that depressed older adults had a lower global quality of life than non-depressed older adults. They went on to say that this link remained constant throughout time and was irrespective of how the quality of life was measured [52].

## Limitations

The limitation of our study is the use of the GDS scale to measure depressive symptoms rather than formal interviews for diagnosing depression, which is thought to be more appropriate for identification and less sensitive to somatic symptoms that could lead to the overestimation of depression. Other limitations include, some of the reports were based on prior events, which can lead to recall bias. Variables like alcohol use, khat chewing, and other substances are more sensitive issues that might lead to social desirability bias. Also, the generalizability of the study might be limited for those who had formal education since the tool (MMSE) is adapted based on the educational level in this setup. In addition, Since income was assessed using the World Bank poverty line, it has limitations such as simple and does not take into account indebtedness, health, education, housing, or public service access. And it does not always fully reflect the differences in subsistence costs between countries. Finally, because of the nature of the cross-sectional study design, it is impossible to establish cause and effect linkages.

## Conclusion

The study conducted in Bahir Dar city shows that more than half of the older adults are suffering from depressive symptoms. An older adult having low educational status, low income, cognitive impairments, a family history of mental illness, and a poor quality of life were all found to be significant predictors of depression in older adults. Because geriatric depression is sometimes unrecognized by clinicians and depressive symptoms are often attributed to the aging process, we recommend that clinicians regularly screen depressive symptoms using standard assessment tools in health care settings and the community. It is preferable to place a greater emphasis on the risk groups identified by this finding.

## Supporting information

S1 File (XLS)

## Abbreviations

AOR: Adjusted odds ratio
CI: Confidence interval
DSM 5: Diagnostic and Statistical Manual 5^th^ text revision
ETB: Ethiopian birr
GDS: Geriatric Depression Scale
HADS: Hospital anxiety and depression scale
HIV/AIDS: Human immune deficiency syndrome
KM: Kilometer
OR: Odds ratio
SPSS: Statistical Packaging for Social Science
USA: United States of American
WHO: World Health Organization

## References

[1] Diagnostic A. statistical manual of mental disorders fifth edition DSM-5. Edisi ke-5 Washington DC: American Psychiatric Association. 2013.

[2] Nair SS, Hiremath S, Ramesh P, Nair SS. Depression among geriatrics: Prevalence and associated factors. Int J Curr Res Rev. 2013;5(8):110–2.

[3] Vanoh D, Shahar S, Yahya HM, Hamid TA. Prevalence and determinants of depressive disorders among community-dwelling older adults: findings from the towards useful aging study. International Journal of Gerontology. 2016;10(2):81–5.

[4] Roh HW, Hong CH, Lee Y, Oh BH, Lee KS, Chang KJ, et al. Participation in physical, social, and religious activity and risk of depression in the elderly: a community-based three-year longitudinal study in Korea. PloS one. 2015;10(7):e0132838. doi: 10.1371/journal.pone.0132838 26172441 PMC4501692

[5] Depression W. Other common mental disorders: global health estimates. Geneva: World Health Organization. 2017:1–24.

[6] Cheng S-T, Siankam B. The impacts of the HIV/AIDS pandemic and socioeconomic development on the living arrangements of older persons in sub-Saharan Africa: A country-level analysis. American journal of community psychology. 2009;44(1):136–47. doi: 10.1007/s10464-009-9243-y 19543825

[7] Organization WH. Mental health: depression in Europe. 2012.

[8] BKA AM, NG MR. Prevalence of cognitive impairment and depression among elderly population in urban Chitradurga. Journal of Preventive Medicine and Holistic Health. 2020;6(1):22–6.

[9] Mulat N, Gutema H, Wassie GT. Prevalence of depression and associated factors among elderly people in Womberma District, north-west, Ethiopia. BMC psychiatry. 2021;21(1):1–9. 33685419 10.1186/s12888-021-03145-x PMC7938572

[10] Padayachey U, Ramlall S, Chipps J. Depression in older adults: prevalence and risk factors in a primary health care sample. South African family practice. 2017;59(2):61–6.

[11] Sharma K, Gupta A, Sharma RC, Mahajan N, Mahajan A, Sharma D, et al. Prevalence and risk factors for depression in elderly North Indians. Journal of Geriatric Mental Health. 2016;3(2):158.

[12] Dao A, Nguyen VT, Nguyen HV, Nguyen LT. Factors associated with depression among the elderly living in urban Vietnam. BioMed research international. 2018;2018. doi: 10.1155/2018/2370284 30596085 PMC6286754

[13] El-Gilany A-H, Elkhawaga GO, Sarraf BB. Depression and its associated factors among elderly: A community-based study in Egypt. Archives of gerontology and geriatrics. 2018;77:103–7. doi: 10.1016/j.archger.2018.04.011 29734054

[14] Subramaniam M, Abdin E, Sambasivam R, Vaingankar JA, Picco L, Pang S, et al. Prevalence of depression among older adults-results from the well-being of the Singapore elderly study. Ann Acad Med Singapore. 2016;45(4):123–33. 27292002

[15] Girma M, Hailu M, Wakwoya A, Yohannis Z, Ebrahim J. Geriatric depression in Ethiopia: prevalence and associated factors. J Psychiatry. 2016;20(1):1000400.

[16] Zou C, Chen S, Shen J, Zheng X, Wang L, Guan L, et al. Prevalence and associated factors of depressive symptoms among elderly inpatients of a Chinese tertiary hospital. Clinical interventions in aging. 2018;13:1755. doi: 10.2147/CIA.S170346 30271130 PMC6145362

[17] Mlaki DA, Asmal L, Paddick SM, Gray WK, Dotchin C, Walker R. Prevalence and associated factors of depression among older adults in rural Tanzania. International Journal of Geriatric Psychiatry. 2021;36(10):1559–66. doi: 10.1002/gps.5584 34018234

[18] Kleisiaris C, Maniou M, Papathanasiou I, Sfiniadaki A, Collaku E, Koutsoumpa C, et al. The prevalence of depressive symptoms in an elderly population and their relation to life situations in home care. Health Science Journal. 2013.

[19] Zhang H-H, Jiang Y-Y, Rao W-W, Zhang Q-E, Qin M-Z, Ng CH, et al. Prevalence of depression among empty-nest elderly in China: a meta-analysis of observational studies. Frontiers in psychiatry. 2020;11:608. doi: 10.3389/fpsyt.2020.00608 32733289 PMC7358371

[20] Charoensakulchai S, Usawachoke S, Kongbangpor W, Thanavirun P, Mitsiriswat A, Pinijnai O, et al. Prevalence and associated factors influencing depression in older adults living in rural Thailand: a cross‐sectional study. Geriatrics & gerontology international. 2019;19(12):1248–53. doi: 10.1111/ggi.13804 31674121

[21] Mirkena Y, Reta MM, Haile K, Nassir Z, Sisay MM. Prevalence of depression and associated factors among older adults at ambo town, Oromia region, Ethiopia. BMC psychiatry. 2018;18(1):1–7. 30336773 10.1186/s12888-018-1911-8 PMC6194620

[22] Goyal A, Kajal K. Prevalence of depression in elderly population in the southern part of Punjab. Journal of family medicine and primary care. 2014;3(4):359. doi: 10.4103/2249-4863.148109 25657943 PMC4311342

[23] Assil S, Zeidan Z. Prevalence of depression and associated factors among elderly Sudanese: a household survey in Khartoum State. EMHJ-Eastern Mediterranean Health Journal, 19 (5), 435–440, 2013. 2013. 24617121

[24] Habte E, Tekle T. Cognitive Functioning among Elders with Symptoms of Depression: The Case of Two Selected Institutionalized Care Centers in Addis Ababa, Ethiopia. Health Science Journal. 2018;12(3).

[25] Seid et al. Population norms for the mini-mental state examination in Ethiopia. Ethiopian medical journal. 2011;49(3). 21991757

[26] da Rocha NS P M B D, Fleck MP. The EUROHIS-QOL 8-item index: comparative psychometric properties to its parent WHOQOL-BREF. Value in Health. 2012;15(3):449–57. doi: 10.1016/j.jval.2011.11.035 22583455

[27] Zhang M, Zhang J, Zhang F, Zhang L, Feng D. Prevalence of psychological distress and the effects of resilience and perceived social support among Chinese college students: Does gender make a difference? Psychiatry Research. 2018;267:409–13. doi: 10.1016/j.psychres.2018.06.038 29960938

[28] Wallace M, Shelkey M. Katz index of independence in activities of daily living (ADL). Urol Nurs. 2007;27(1):93–4. 17390935

[29] Hailemariam H, Singh P, Fekadu T. Evaluation of mini nutrition assessment (MNA) tool among community dwelling elderly in urban community of Hawassa city, Southern Ethiopia. BMC Nutrition. 2016;2(1):1–6.

[30] McRee B, Babor TF, Lynch ML, Vendetti JA. Reliability and validity of a two-question version of the World Health Organization’s alcohol, smoking and substance involvement screening test: The ASSIST-FC. Journal of Studies on Alcohol and Drugs. 2018;79(4):649–57. 30079882

[31] Hodgson R, Alwyn T, John B, Thom B, Smith A. The FAST alcohol screening test. Alcohol and alcoholism. 2002;37(1):61–6. doi: 10.1093/alcalc/37.1.61 11825859

[32] Bank W. Decline of global extreme poverty continues but has slowed. Accessed on. 2019;28.

[33] Sarkar S, Kattimani S, Roy G, Premarajan K, Sarkar S. Validation of the Tamil version of short form Geriatric Depression Scale-15. Journal of neurosciences in rural practice. 2015;6(03):442–1446. doi: 10.4103/0976-3147.158800 26167040 PMC4481811

[34] Gautam R, Houde S. Geriatric depression scale for community-dwelling older adults in Nepal. Asian j gerontol Geriatr. 2011;6(2):93–9. 10.1016/j.archger.2010.06.007 20598380

[35] Yesavage JA, Brink TL, Rose TL, Lum O, Huang V, Adey M, et al. Development and validation of a geriatric depression screening scale: a preliminary report. Journal of psychiatric research. 1982;17(1):37–49. doi: 10.1016/0022-3956(82)90033-4 7183759

[36] Sadock BJ, Sadock VA. Kaplan and Sadock’s synopsis of psychiatry: Behavioral sciences/clinical psychiatry: Lippincott Williams & Wilkins; 2011.

[37] folstein MF S.E AND McHugh P.R. mini-mental statue: A practical method for grading the cognitive state o patients for the clinician. journal of psychiatry research. 1975;12(3):189–98. 10.1016/0022-3956(75)90026-6 1202204

[38] Azad A, Mohammadinezhad T, Taghizadeh G, Lajevardi L. Clinical assessment of activities of daily living in acute stroke: Validation of the Persian version of Katz Index. Medical journal of the Islamic Republic of Iran. 2017;31:30. doi: 10.18869/mjiri.31.30 29445659 PMC5804429

[39] Leal MCC, Apóstolo JLA, Mendes AMdOC, Marques APdO. Prevalence of depressive symptoms and associated factors among institutionalized elderly. Acta Paulista de Enfermagem. 2014;27:208–14.

[40] Goud AA, Nikhade NS. Prevalence of depression in older adults living in old age home. IAIM. 2015;2(11):1–5.

[41] Babatsikou F, Konsolaki E, Notara V, Kouri M, Zyga S, Koutis C. Depression in the elderly: a descriptive study of Urban and Semi-Urban Greek population. International Journal of Caring Sciences. 2017;10(3):1286–95.

[42] Buvneshkumar M, John K, Logaraj M. A study on prevalence of depression and associated risk factors among elderly in a rural block of Tamil Nadu. Indian journal of public health. 2018;62(2):89. doi: 10.4103/ijph.IJPH_33_17 29923530

[43] Ekram M ES , Hany H ED , Eman ET , Al Shymaa M L . Depression among elderly in Beni Suef city [Upper Egypt]. 2009.

[44] Odejimi O, Tadros G, Sabry N. A systematic review of the prevalence of mental and neurocognitive disorders amongst older adults’ populace in Egypt. Middle East Current Psychiatry. 2020;27(1):1–12.

[45] Cho SM, Saw YM, Saw TN, Than TM, Khaing M, Khine AT, et al. Prevalence and risk factors of anxiety and depression among the community-dwelling elderly in Nay Pyi Taw Union Territory, Myanmar. Scientific Reports. 2021;11(1):1–9. 33963225 10.1038/s41598-021-88621-w PMC8105404

[46] Sousa RDd, Rodrigues AM, Gregório MJ, Branco JDC, Gouveia MJ, Canhão H, et al. Anxiety and depression in the Portuguese older adults: Prevalence and associated factors. Frontiers in Medicine. 2017;4:196. doi: 10.3389/fmed.2017.00196 29209612 PMC5702006

[47] McCall NT, Parks P, Smith K, Pope G, Griggs M. The prevalence of major depression or dysthymia among aged Medicare Fee‐for‐Service beneficiaries. International journal of geriatric psychiatry. 2002;17(6):557–65. doi: 10.1002/gps.642 12112180

[48] Ismail Z, Elbayoumi H, Fischer CE, Hogan DB, Millikin CP, Schweizer T, et al. Prevalence of depression in patients with mild cognitive impairment: a systematic review and meta-analysis. JAMA psychiatry. 2017;74(1):58–67. doi: 10.1001/jamapsychiatry.2016.3162 27893026

[49] Steffens DC, Potter G. Geriatric depression and cognitive impairment. Psychological medicine. 2008;38(2):163–75. doi: 10.1017/S003329170700102X 17588275

[50] Lebedeva AK, Westman E, Borza T, Beyer MK, Engedal K, Aarsland D, et al. MRI-based classification models in prediction of mild cognitive impairment and dementia in late-life depression. Frontiers in aging neuroscience. 2017;9:13. doi: 10.3389/fnagi.2017.00013 28210220 PMC5288688

[51] Sadock BJ. Kaplan & Sadock’s synopsis of psychiatry: behavioral sciences/clinical psychiatry. 2007.

[52] Sivertsen H, Bjørkløf GH, Engedal K, Selbæk G, Helvik A-S. Depression and quality of life in older persons: a review. Dementia and geriatric cognitive disorders. 2015;40(5–6):311–39. doi: 10.1159/000437299 26360014

