# Supplementary material for: Depression and associated factors among older adults in Bahir Dar city administration, Northwest Ethiopia, 2020: Cross-sectional study
Source: PLoS One. 2022 Aug 23;17(8):e0273345. doi: 10.1371/journal.pone.0273345 (PMC9397869; doi:10.1371/journal.pone.0273345)
Supplement: Attachment — Submitted filename: Response to Reviewers.docx [file pone.0273345.s003.docx]

**Response to editor and reviewers**

Please review your paper and make the manuscript clear and reader-friendly with following concerns with other reviewers comments:
1. Abstract results section line 34 CI 53.2, 62.6 should be consistent with other Cis

**Response:** We corrected as recommended.
2. Make an abstract conclusion very specific based on your findings and provide pragmatic suggestions which are very vague now. Provide at least 5 appropriate keywords.

**Response:** We revised it extensively. See the manuscript. Thank you dear editor to give achance to see the manuscript again.
3. Line 74 writes in word one instead of 1 million.

**Response:** Corrected.
4. In the methods and materials section starts with the research design followed by the study population (remove line 122) and merges the study population with inclusion and exclusion criteria, study sites, sample size, and sampling techniques,..

**Response:** We corrected it as per your recommendation.
5. line 207 removes processing and keeps only data analysis

**Response:** Thank you, we corrected as per a recommendation.
6. Line 219 writes down Ethical consideration

**Response:** We revised it accordingly.
7. Line 245 Results

**Response:** We corrected it.
8. Line 247-258 presents very key findings in only % and refer to Table 1 for details.

**Response:**We corrected it.
9. Present mean/median age with SD or IQR

**Response:** We present it as recommended.
10. Review table 1 educational status variables and present constantly i.e 5-8th vs 9-12^th^

**Response:** Thank you, we corrected it.
11. Table 1 presents the mean/median monthly income with SD/IQR

**Response:** Thank you, we corrected it as per recommendation.
12. Line 261-267 review findings and present key findings with % and recommend Table 2 for the details.

**Response:** Thank you, we revised it.
13. Review the lines 271-273 as per above.

**Response:** Again we thank you, and revised as you recommended.
14. Line 285 headings and 290 should merge and start with prevalence instead of separate headings and also make consistent terminology of the figure headings older adults instead of elderly

**Response:** We revised it as per your recommendation. See the revised manuscript.
15. Line 280 - 307 present only key findings

**Response:** Thank you, dear editor, in this we tried to show the important points. If specific things to be revised we are welcome**.**
16. Line 307 correct error is showing not sure what is written.

**Response:** In line 307, we found this value (AOR: 2.78, 95% CI: 1.74–4.46), which was written correctly in table 4. If any issues again we are welcome to revise it.

**Response to reviewers**
1. Prevalence calculation.
The authors have stated that they calculated the prevalence of depression as follows:
Prevalence= (Total cases)/ (Total population) x 100, P=245/423 x 100 = 57.9%
This is prevalence of depression among the older adults screened for depression in Bahir Dar City not the prevalence of depression among older adults in Bahir Dar City.
The authors stated that the total population of older adults aged 60 years and above in Bahir Dar City is 11,034 (5003 male and 6031 females). The correct approach would be estimating the number of cases of depression in each sub-city, adding the cases and then diving by the total population ( 11,034).
**Response:** Thank you dear reviewer.

**Prevalence is calculated as follows;**

1. To estimate prevalence, researchers randomly select a sample (smaller group) from the entire population they want to describe.
2. For a representative sample, prevalence is the number of people in the sample with the characteristic of interest, divided by the total number of people in the sample.

-If we calculate by including the total population (11,034), we are saying we didn’t need a sample size calculation. Analysis should be conducted based on collected data or sample size included in the study (423). So we put it as it is.
2. Informed consent.
In their response to reviewers’ comments, the authors stated that almost all older adults had severe cognitive impairment. However, in the manuscript only 42% had cognitive impairment. It is not clear if these older adults with severe cognitive impairment had capacity to provide a valid informed consent. This should be clarified.

**Response:** Thank you, dear reviewer. Previously we respond only 8(1.9%) had **severe** cognitive impairment. Informed (written) consent was obtained from each study participant. The study participants were also provided with information about the objectives and expected outcomes of the study. Information obtained from individual participants was kept secure and confidential. We had given time to ask questions repeatedly for better thinking, understanding, and responding to the questions.
3. Abstract
The conclusion does not reflect what the authors have studied. For example, the study did not look at supportive therapy for the treatment of depression in this population.

**Response:** We revised it accordingly as per your recommendation. Again, thank you!
4. Other issues.
There are several grammatical errors in this manuscript. Eg lines 172-176. These should to be corrected.

**Response:** We revised it accordingly. See the main manuscript.
Line ‘hard time’, use scholastic language

**Response:** Dear reviewer, thank you for your recommendation to revise it. We replaced it with “more difficult”
Line 5 and 6, empty space
Lines 11-19, empty space
Lines 47 -49, empty space
Lines 96-105 , empty space
Line 206, empty space
Lines 229-244 empty space
Lines 282- 284 empty space
Lines 308- 311 empty space
Lines 318, 395, 405, 438-440 empty space

**Response:** We corrected all empty spaces.
-References
Several references eg no 17, 29, 32 are incorrectly cited. This should be corrected.

**Response:** We corrected it as per recommendation.

**Regards,**

**Tamrat Anbesaw( Corresponding Author)**
