# Supplementary material for: Depression and associated factors among older adults in Bahir Dar city administration, Northwest Ethiopia, 2020: Cross-sectional study
Source: PLoS One. 2022 Aug 23;17(8):e0273345. doi: 10.1371/journal.pone.0273345 (PMC9397869; doi:10.1371/journal.pone.0273345)
Supplement: Attachment — Submitted filename: Response to editor and reviewers revised.docx [file pone.0273345.s004.docx]

**Response to editor and reviewers**

Thank you dear Editor for giving a chance to revise it again. We have seen the manuscript critically and revised as per recommendation.

**Question: Could you please remove line 94 and 95 Source Population and All older adults who live in Bahir Dar city administration which you have talked in line 83.**

**Response: We removed it as recommendation.**
